# Mapping the Microstructure and Striae of the Human Olfactory Tract with Diffusion MRI

**DOI:** 10.1523/JNEUROSCI.1552-21.2021

**Published:** 2022-01-05

**Authors:** Shiloh L. Echevarria-Cooper, Guangyu Zhou, Christina Zelano, Franco Pestilli, Todd B. Parrish, Thorsten Kahnt

**Affiliations:** ^1^Department of Neurology, Northwestern University, Feinberg School of Medicine, Chicago, Illinois 60611; ^2^The Graduate School, Northwestern University Interdepartmental Neuroscience (NUIN), Evanston, Illinois 60208; ^3^Department of Psychology, Northwestern University, Weinberg College of Arts and Sciences, Evanston, Illinois 60208; ^4^Department of Psychology, The University of Texas at Austin, Austin, Texas 78712; ^5^Center for Perceptual Systems, The University of Texas at Austin, Austin, Texas 78712; ^6^Department of Radiology, Northwestern University, Chicago, Illinois 60611

**Keywords:** diffusion MRI, human, odor perception, olfaction, olfactory tract, tractography

## Abstract

The human sense of smell plays an important role in appetite and food intake, detecting environmental threats, social interactions, and memory processing. However, little is known about the neural circuity supporting its function. The olfactory tracts project from the olfactory bulb along the base of the frontal cortex, branching into several striae to meet diverse cortical regions. Historically, using diffusion magnetic resonance imaging (dMRI) to reconstruct the human olfactory tracts has been prevented by susceptibility and motion artifacts. Here, we used a dMRI method with readout segmentation of long variable echo-trains (RESOLVE) to minimize image distortions and characterize the human olfactory tracts *in vivo*. We collected high-resolution dMRI data from 25 healthy human participants (12 male and 13 female) and performed probabilistic tractography using constrained spherical deconvolution (CSD). At the individual subject level, we identified the lateral, medial, and intermediate striae with their respective cortical connections to the piriform cortex and amygdala (AMY), olfactory tubercle (OT), and anterior olfactory nucleus (AON). We combined individual results across subjects to create a normalized, probabilistic atlas of the olfactory tracts. We then investigated the relationship between olfactory perceptual scores and measures of white matter integrity, including mean diffusivity (MD). Importantly, we found that olfactory tract MD negatively correlated with odor discrimination performance. In summary, our results provide a detailed characterization of the connectivity of the human olfactory tracts and demonstrate an association between their structural integrity and olfactory perceptual function.

**SIGNIFICANCE STATEMENT** This study provides the first detailed *in vivo* description of the cortical connectivity of the three olfactory tract striae in the human brain, using diffusion magnetic resonance imaging (dMRI). Additionally, we show that tract microstructure correlates with performance on an odor discrimination task, suggesting a link between the structural integrity of the olfactory tracts and odor perception. Lastly, we generated a normalized probabilistic atlas of the olfactory tracts that may be used in future research to study its integrity in health and disease.

## Introduction

Human olfaction supports many important cognitive and behavioral functions, including food-intake, social interactions, memory, and detecting threats in the environment (Gottfried, 2010; McGann, 2017). Despite its importance, much of our knowledge about the connectivity of olfactory bulb afferents is inferred from work in nonhuman animals. Postmortem studies in humans suggest that the olfactory tracts are comprised of parallel afferents that split into three separate striae (lateral, medial, and intermediate) before meeting primary olfactory cortex, but their precise targets remain difficult to identify (Rose, 1927; Mark et al., 1994; Allison, 1954; Kavoi and Jameela, 2011). In rats and mice, axon tracing reveals projections to the anterior olfactory nucleus (AON), anterior and posterior piriform cortices, the olfactory tubercle (OT), the amygdala (AMY), periamygdaloid cortex, and lateral entorhinal cortex (EC; White, 1965; Haberly and Price, 1978a,b; Scott et al., 1980; Schwob and Price, 1984; Miyamichi et al., 2011). In macaque monkeys, projections identified with axon tracing methods appear to be highly conserved and innervate homologous primary olfactory regions, but connectivity to the EC is confined only to its most rostral aspect (Carmichael et al., 1994). Homologous cortical regions have been identified in humans (Uyematsu, 1921; Rose, 1927; Crosby and Humphrey, 1941; Allison, 1954; Kavoi and Jameela, 2011), including the AON, frontal piriform cortex (FPC), temporal piriform cortex (TPC), OT, AMY, and EC. Allison (1954) identified the striae of the postmortem human olfactory tracts with silver staining, and concluded that they reached each of these regions with the exception of EC. However, precise replication of these findings using *in vivo* methods is still needed.

*In vivo* investigations of the human olfactory tracts have only recently become possible with innovations in diffusion magnetic resonance imaging (dMRI; Skorpil et al., 2011; Fjaeldstad et al., 2017; Milardi et al., 2017). However, several limitations have so far prevented a comprehensive mapping of their connectivity. First, magnetic susceptibility differences between brain tissue and air in the sinus cavities cause severe artifacts, warping the final image and obscuring the olfactory tracts. Second, dMRI scans are particularly sensitive to head motion. Third, the branching and highly curved olfactory tract striae pose problems for the traditional diffusion tensor model, which cannot model multiple fiber orientations within a single voxel (Tournier et al., 2007, 2012).

In the present study, we sought to overcome these challenges by using recent advances in dMRI technology. Most importantly, we used a method with readout segmentation of long variable echo-trains (RESOLVE) to achieve short echo times (TEs), allowing high-resolution scanning with relatively few susceptibility artifacts (Porter and Heidemann, 2009). We also used customized head stabilizers to “head-fix” participants during scanning (Power et al., 2019). Finally, to discern the branching striae, we performed probabilistic tractography using the constrained spherical deconvolution (CSD) model, capable of fitting multiple fiber orientations within each voxel (Tournier et al., 2007, 2012).

Using these optimized methods, we have identified the three striae of the olfactory tracts and characterized their connectivity with primary olfactory cortex in 25 healthy human subjects. Further, we found a correlation between the microstructural integrity of the olfactory tracts and olfactory perceptual function. These results provide novel insight regarding human olfactory tract connectivity, which has historically been difficult to discern. They also provide the first step toward investigating *in vivo* microstructure-function relationships in the human olfactory system, which may be useful for studying olfactory tissue integrity in clinical populations. Specifically, olfactory dysfunction may serve as an early harbinger of neurodegenerative diseases such as Parkinson's (Witt et al., 2009; Fullard et al., 2017) or Alzheimer's disease (Peters et al., 2003; Murphy, 2019), and in demyelinating diseases such as multiple sclerosis (Lucassen et al., 2016; Carotenuto et al., 2019). Identifying specific patterns of tissue degeneration in conjunction with olfactory perceptual testing may help dissociate different degenerative diseases in their prodromal stages.

## Materials and Methods

### Subjects

A total of 27 right-handed subjects (14 male and 13 female; age: mean 25.76 ± SD 4.01 years), with no neurologic disorders, psychiatric disorders, or MRI contraindications, were enrolled in this study. Two subjects, both males, were excluded from final analyses because they did not complete the MRI scanning protocol. The study was approved by the Northwestern IRB (STU00098371), and all subjects gave written informed consent for participation.

### Study design

**Figure 1.**
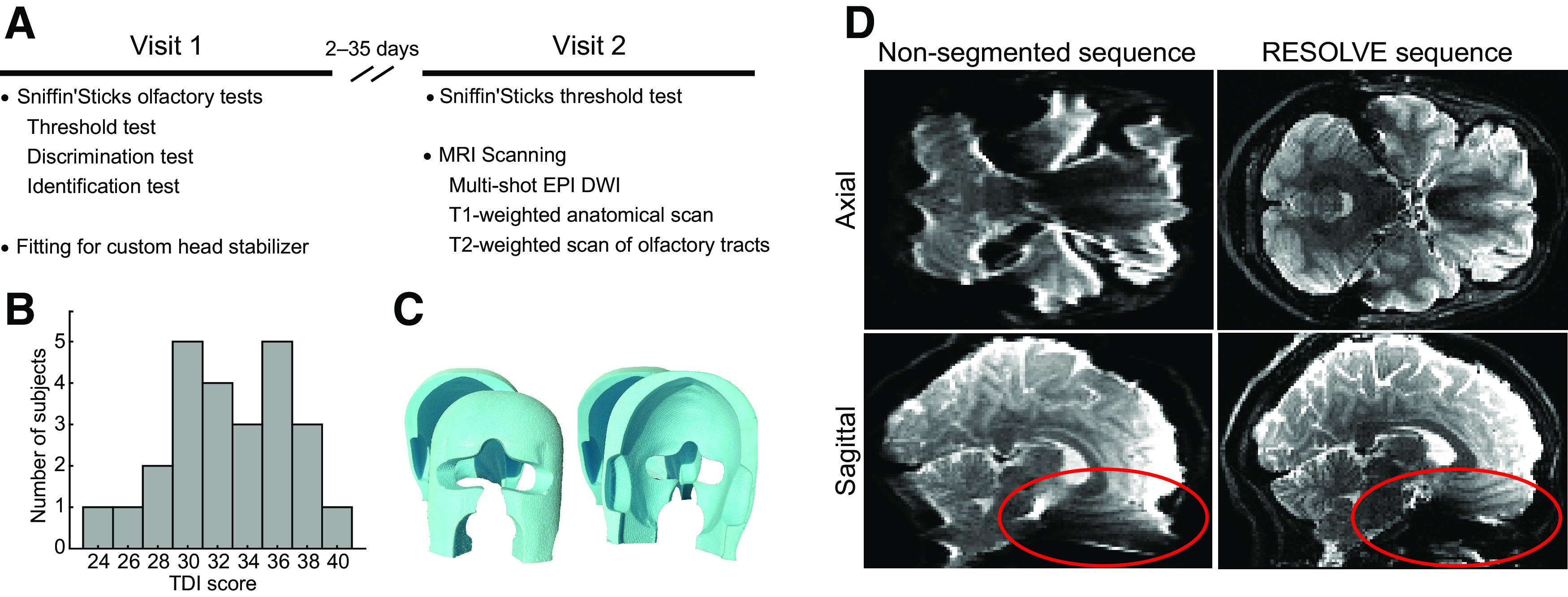
***A***, Study timeline. DWI = diffusion weighted imaging. ***B***, Histogram of the summed TDI (Threshold, Discrimination, and Identification) scores across subjects. ***C***, Example of a customized 3D-milled head stabilizer for preventing head motion during MRI scanning. ***D***, Comparison of susceptibility artifacts and blurring at 1.5-mm isotropic resolution between a nonsegmented Single-Shot Echo Planar Imaging (SS-EPI) sequence and the multi-shot Readout Segmentation of Long Varaible Echo trains (RESOLVE) sequence (collected from the same pilot subject). Note that severe artifacts present in orbitofrontal regions in the nonsegmented EPI images are absent in the RESOLVE images (red).

Subjects visited the lab two times (Fig. 1*A*). During visit 1, they completed three olfactory perceptual tests (threshold, discrimination, and identification) and were fitted for a personalized head stabilizer. During visit 2, participants repeated the olfactory threshold test, and underwent MRI scanning. Visit 1 and visit 2 were separated by 2–35 d (mean 15.68 ± SD 9.51).

### Olfactory perceptual testing

During visit 1, subjects underwent olfactory perceptual testing using the Sniffin' Sticks threshold (n-butanol), discrimination, and identification tests (Rumeau et al., 2016), administered in the listed order. During visit 2, subjects repeated the olfactory threshold test, and the two threshold scores were averaged. Scores on each test range from 0 (worst) to 16 (best), with anosmic thresholds at scores of T = 1.0, D = 8, and I = 8. All subjects scored above anosmic thresholds for all three tests. We computed the composite threshold + discrimination + identification (TDI) score by adding the mean threshold score, the discrimination score, and the identification score (Fig. 1*B*).

### Personalized head stabilizers

Subjects wore personalized head stabilizers to prevent motion for the duration of MRI scanning (Power et al., 2019). 3D renderings of each subject's face and head were created using a handheld camera and the Caseforge iOS application. The head stabilizers were 3D-milled to fit the subject's face and head on the inside and the shape of the MRI scanner coil on the outside. An example is shown in Figure 1*C*.

### MRI data acquisition

During visit 2, subjects underwent MRI scanning on a 3T Siemens Prisma scanner with a 64-channel head-neck coil. We collected a set of diffusion-weighted images, a T1-weighted image, and a T2-weighted image. Subjects wore their customized head stabilizers for the duration of the scans.

We used a high-resolution (1.5 mm isotropic) RESOLVE dMRI scan with seven readout segments (Porter and Heidemann, 2009) to collect the diffusion-weighted images. This sequence is different from typical single-shot echo planar imaging (SS-EPI) techniques in that it splits data collection into seven segments in the read-out direction and re-excites the tissue before each segment with a new radio frequency pulse. The readout segments are combined in the end to produce the full image. The shorter readout segment allows for a shorter TE than is possible in SS-EPI sequences. However, it takes more time to acquire a complete dataset, based on the number of segments. We also included a navigator echo to monitor between-segment motion, so that volumes were re-acquired if the motion was excessive (Porter and Heidemann, 2009). In addition, we used simultaneous multi-slice acquisition (Nunes et al., 2006) to allow for improved spatial coverage required when using such small voxels. This sequence was designed based on extensive pilot testing to provide high-resolution images with reduced blurring, and largely free of susceptibility artifacts compared with conventional SS-EPI techniques (Fig. 1*D*). Imaging parameters were as follows: 92 slices; field of view (FoV) = 240 mm; matrix size = 240 × 240 × 138 mm; 90 diffusion-weghted directions at b = 1000 s/mm^2^; 12 interspersed b0 volumes; phase encoding = A > P; TE1 (image echo) = 61 ms; TE2 (navigator echo) = 98 ms; repetition time (TR) = 6250 ms; flip angle = 180°; bandwidth = 897 Hz/Px, multiband factor = 2. The scan time for this RESOLVE dMRI sequence was ∼1 h and 30 min. An oblique slice angle (∼30° relative to the AC–PC plane) was used to further reduce susceptibility artifacts (Weiskopf et al., 2006).

The parameters for the two anatomic scans were as follows: T1-weighted, 1.0 mm isotropic, TE = 2.94 ms, TR = 2300 ms, flip angle = 9°, FoV = 256 mm, matrix size = 256 × 256 × 176 mm; phase encoding = A > P, bandwidth = 240 Hz/Px; T2-weighted (Siemens ZOOMit protocol), 0.5 mm isotropic, TE = 125 ms, TR = 1000 ms, flip angle = 100°, FoV = 160 mm, matrix size = 82 × 160 × 72 mm, phase encoding = A > P, bandwidth = 256 Hz/Px. The T2-weighted image covered the ventral frontal lobes and temporal poles, including the olfactory bulbs, orbitofrontal cortex, and lengths of the olfactory tracts. The scan duration was 5 min for the T1-weighted image and 7 min for the T2-weighted image.

### MRI data preprocessing

All MRI data were converted to the Nifti file type using MRIcron's dcm2niix function (Li et al., 2016). The diffusion MRI data were corrected for motion and eddy current artifacts using FSL's function, eddy_openmp (Smith et al., 2004; Woolrich et al., 2009; Jenkinson et al., 2012). The T1-weighted and T2-weighted images were co-registered to the native diffusion space using SPM12 (SPM12 Software, 2014). All diffusion model fitting and tractography were performed in the native diffusion space to prevent registration-related errors in the alignment of the b-vectors with the diffusion-weighted data. MRtrix2 functions were used to fit the tensor model (dwi2tensor), create FA (tensor2FA) and eigenvector maps (tensor2vector), estimate the fiber response function for use in spherical deconvolution (estimate_response), and fit the CSD model (csdeconv; Lmax = 8; Tournier et al., 2007, 2008, 2012). The MRtrix3 function tensor2metric was used to generate mean diffusivity (MD) maps based on the estimated diffusion tensors (Basser et al., 1994; Westin et al., 1997; Tournier et al., 2019). The CSD model was used to perform probabilistic fiber tractography, using the MRtrix2 function streamtrack SD_PROB (Tournier et al., 2012), to delineate the paths traversed by the olfactory tracts (see below, Probabilistic tractography).

### Regions of interest (ROIs)

**Figure 2.**
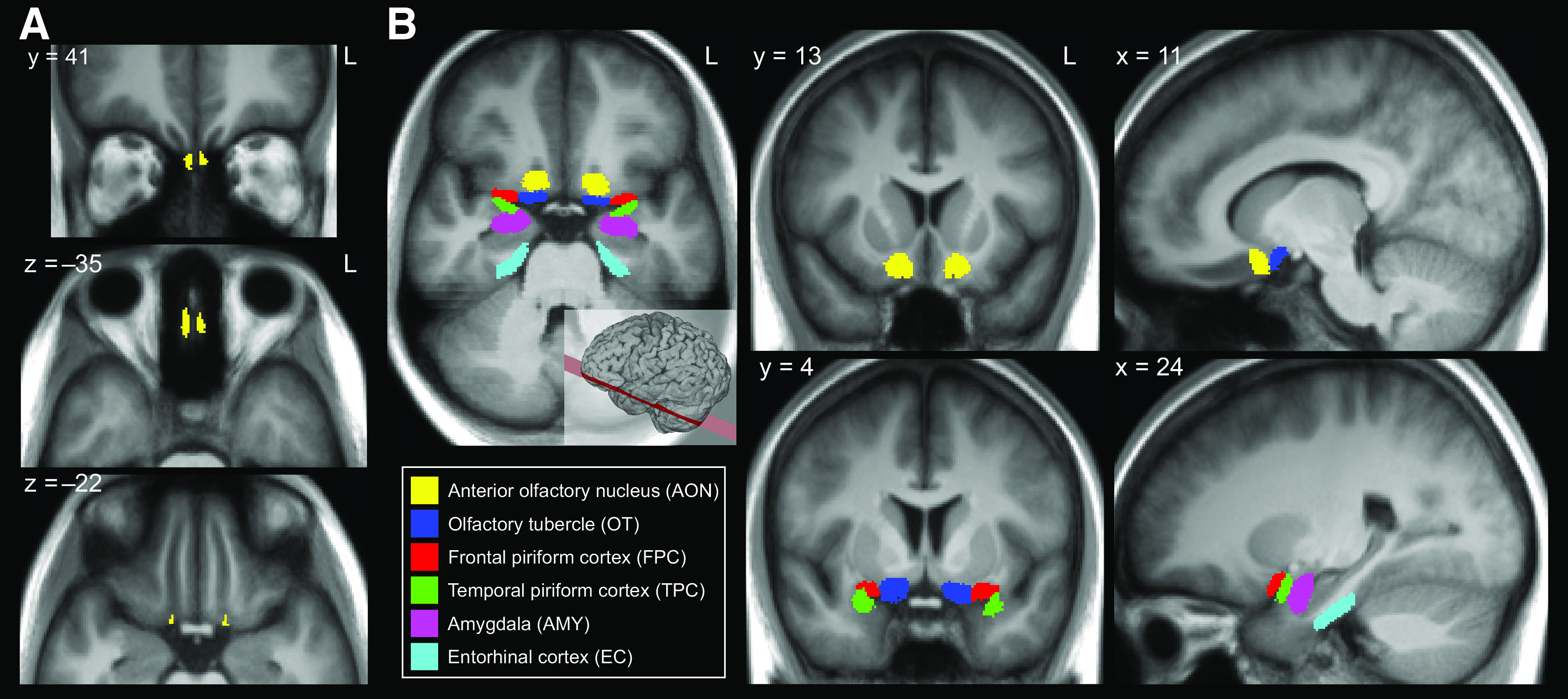
Atlases of the Regions of Interest in MNI space. ***A***, Seed regions of the olfactory bulb (top and middle) and midpoint of the olfactory tract (bottom) used for segmentation of the olfactory tracts. ***B***, Masks of the primary olfactory regions that were used as inclusionary regions investigated for connectivity with the olfactory tracts. Only voxels overlapping in >20% of subjects are shown for illustration. Masks are overlaid on a mean image of all subjects' MNI-normalized T1 images.

Olfactory ROIs were defined for use in tractography segmentation (Fig. 2) using ITK-SNAP (Yushkevich et al., 2006). The ROIs were drawn for the left and right hemispheres separately on each individual's anatomic images.

The olfactory bulbs were outlined on each individual's 0.5-mm resolution T2-weighted image. Using both the 0.5-mm resolution T2-weighted and 1.0-mm resolution T1-weighted images, ROIs were placed in a midpoint region of the olfactory tracts in both hemispheres. This midpoint ROI was placed in the olfactory sulcus, anterior to the position of the olfactory trigones, and posterior and superior to the level where the optic nerves traverse below the olfactory sulci. In some subjects, a portion of the olfactory tract is visible at this location in the anatomic images and in the FA maps.

Several cortical and subcortical ROIs were defined based on established targets of the olfactory tracts in rodents and nonhuman primates (White, 1965; Haberly and Price, 1978a,b; Carmichael et al., 1994; Miyamichi et al., 2011). These regions included the AON, OT, FPC, TPC, AMY, and EC. These regions were defined for each subject, separately in the left and right hemispheres based on a published atlas (Mai et al., 2015), architectonic studies (Öngür and Price, 2000; Ongür et al., 2003), and the results of an olfactory functional network study (Zhou et al., 2019). To generate probabilistic atlases for these olfactory ROIs, each subject's ROIs were normalized to MNI space and binarized, and the normalized ROIs were averaged across subjects, resulting a probability value for each voxel. These ROI atlases are available on NeuroVault (https://neurovault.org/collections/ZTCWDMII/) and BrainLife (https://brainlife.io/project/5ac2a489e182730027c55588).

### Probabilistic tractography

The olfactory tracts were defined for each subject, separately in each hemisphere, with probabilistic tractography based on the CSD model, using the streamtrack SD_PROB algorithm (Lmax = 8, FA threshold = 0.1, curvature threshold = 1.5 mm) from MRtrix2 (Tournier et al., 2007, 2008, 2012). Three sets of fiber groups were generated in each hemisphere (for details, see Results). For each fiber group, probabilistic tractography continued until 1000 streamlines were generated meeting the defined conditions. Brain masks were not used to constrain tracking, since most brain masking algorithms exclude the olfactory bulbs and probable locations of the olfactory tracts, because of the signal quality of conventional diffusion-weighted images. Fiber groups were then cleaned using the dtiCleanFibers and AFQ_removeFiberOutliers functions from Vistasoft and the Automated Fiber Quantification (AFQ) package (Yeatman et al., 2012; Pestilli et al., 2014). These functions remove any streamlines that are >4 SDs longer than the mean streamline length, or that are >4 SDs outside of the mean Gaussian distance from the “core” of the fiber tract, as defined in Yeatman et al. (2012).

In most subjects, the olfactory bulbs could not be continuously linked to cortex because of a small area of signal drop out near the sphenoid sinus. In these subjects, we used the MATLAB function cscvn (version R2020b) to produce natural cubic spline curves (Lee, 1989) to interpolate the path of the olfactory tracts across this gap, separately for each subject in each hemisphere. The five most posterior points (1 mm) of fiber group 1 and the five most anterior points (1 mm) of fiber group 2 were excluded, since the streamlines tended to splay out away from the core of the fiber tract near the ends. The next five most posterior points (1 mm) of the streamlines in fiber group 1 and the next five most anterior points (1 mm) of the streamlines in fiber group 2 were used as control points for interpolation.

In all subjects, the midpoint seeding regions of the olfactory tracts were linked to primary olfactory cortical ROIs via continuous streamlines, in fiber groups 2 and 3. These fiber groups were used to analyze connectivity between the olfactory tracts and individual cortical ROIs. Connectivity was noted as present if streamlines existed connecting the seeding region in the olfactory tracts with the cortical ROI in question. Connection density values were calculated for each connection to describe the strength of each connection, defined as the number of streamlines connecting the seeding region to each cortical ROI, divided by the volume (mm^3^) of that cortical ROI.

### Generation of the olfactory tract atlas

We created binary masks in each subject's native space to index voxels traversed by the olfactory tracts, using the cleaned, interpolated, and combined fiber groups. We then normalized these masks into Montreal Neurological Institute (MNI) space using SPM12 (SPM12 Software, 2014) with deformation fields estimated based on the T1-weighted images. We averaged the masks in MNI space across subjects to create a probabilistic atlas of the olfactory tracts, where each voxel's value between 0 and 1 reflects the proportion of subjects in which olfactory tract streamlines were present at that position. We truncated the posterior boundary of the olfactory tract atlases at MNI Y = –3, just posterior to the point where the lateral and medial striae enter cortex. The anterior boundary is located at MNI Y = 53, at the anterior edge of the olfactory bulbs. This atlas is publicly available on NeuroVault (https://neurovault.org/collections/ZTCWDMII/) and on BrainLife (https://brainlife.io/project/5ac2a489e182730027c55588).

### Diffusion microstructure profiles of the olfactory tracts

We conducted analyses of local olfactory tract microstructural characteristics in individual subjects, using our probabilistic olfactory tract atlas. Fractional anisotropy (FA) and mean diffusivity (MD) estimates in a white matter tract of interest are known to be affected by partial volume effects with surrounding anatomy, inhomogeneities in the magnetic field, and noise. Local measures of FA and MD can thus be plotted along a white matter tract to account for these effects, and this method has been shown to produce replicable characteristic curves for specific white matter tracts across healthy subjects (Yeatman et al., 2012). Here, we used a similar approach in the olfactory tracts.

We first divided the olfactory tract atlas into eight equal-spaced anterior-posterior segments (width = 6.25 mm) in each hemisphere, in MNI space. We reasoned that averaging FA and MD measures within segments of this size would help to reduce noise while preserving local information about field inhomogeneities and neighboring anatomic features. We then then transformed the segmented masks into each subjects' native diffusion space, using SPM12 (SPM12 Software, 2014) with inverse deformation fields estimated based on the T1-weighted images. For each subject, we calculated voxel-wise values for FA and MD in each segment and weighted these values by the probability values in the olfactory tract atlas. We then calculated the mean of the weighted FA and MD measures within each segment, for each subject.

### Statistical analysis

To test correlations between microstructure measures and olfactory perceptual ability, we regressed the FA and MD values in each of the eight segments (averaged across hemispheres) against scores on each of the Sniffin' Sticks tests (threshold, discrimination, and identification). We controlled for potential effects of age and sex, by including these variables as covariates in multiple linear regression models. Bonferroni correction was used to correct for multiple comparisons (eight segments × three measures).

## Results

Healthy subjects [*N* = 25, 13 female, age 24.98 ± 4.38 (mean ± SD) years] participated in olfactory perceptual testing and MRI scanning (Fig. 1*A*). Summed Sniffin' Sticks TDI scores are shown in Figure 1*B*. During MRI scanning, subjects wore individualized head stabilizers (Fig. 1*C*) to prevent motion. MRI scanning included 1.0-mm isotropic T1-weighted and 0.5-mm isotropic T2-weighted structural MRI scans, used to identify anatomic ROIs, and 1.5-mm isotropic dMRI RESOLVE scans. We chose the dMRI RESOLVE sequence based on extensive pilot testing in our lab to produce high-resolution images with reduced blurring, and largely free of susceptibility artifacts compared with conventional SS-EPI dMRI techniques (Fig. 1*D*).

### Tractography and connectivity results

We reconstructed olfactory tract streamlines in each subject using probabilistic tractography based on the CSD model (Tournier et al., 2007, 2012). In each hemisphere, we defined ROIs for each individual subject, including the olfactory bulb, several primary olfactory cortical regions (including the AON, FPC, TPC, OT, AMY, and EC), and a midpoint region of the olfactory tract located in the olfactory sulcus (Fig. 2). We generated three sets of fiber groups in each hemisphere with the following conditions: (1) streamlines were seeded from the olfactory bulbs; (2) streamlines were seeded from the olfactory tract midpoint ROI, and olfactory cortical regions including the AON, FPC, TPC, OT, AMY, and EC were defined as inclusionary ROIs; (3) streamlines were seeded from the cortical ROIs listed in the second condition, and the olfactory tract midpoint ROI was defined as an inclusionary ROI. In all three conditions, exclusionary ROIs were placed to prevent streamlines from crossing the midline or entering the optic nerves, gyrus rectus, orbitofrontal cortex, or the surrounding cerebrospinal fluid. Fiber groups were cleaned to remove noisy and erroneous streamlines (see Materials and Methods), and the resulting fiber groups contained (mean ± SD) 952.64 ± 24.98, 879.64 ± 40.05, and 853.60 ± 37.73 streamlines in the left hemisphere for fiber groups 1–3, respectively. The number of streamlines was 963.64 ± 23.49, 873.48 ± 30.75, and 864.92 ± 31.91 for groups 1–3 in the right hemisphere. Fiber groups 2 and 3 were used to evaluate the connectivity of the olfactory tracts with primary olfactory cortex.

**Figure 3.**
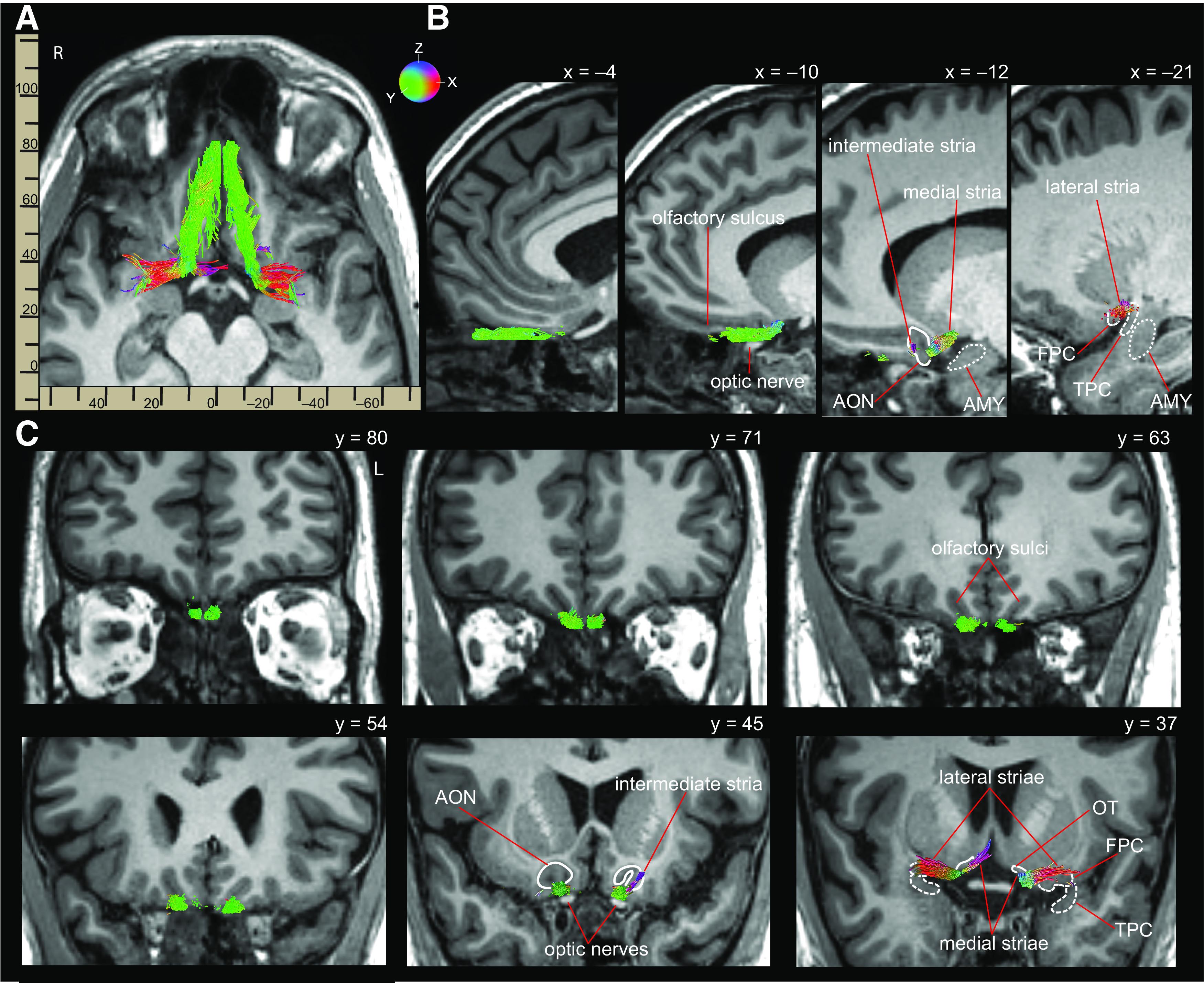
Continuous streamlines connecting the olfactory bulbs with primary olfactory cortex in one subject (Table 2, row 4), overlaid on the subject's T1 image. ***A***, 3D fiber groups overlaid on an axial slice. ***B***, Sagittal views of the fiber groups in the left hemisphere. ***C***, Coronal views of the fiber groups indicating the trajectory of the olfactory tracts from bulb (y = 80) to the intermediate stria (left hemisphere, y = 45), and the medial (right hemisphere) and lateral striae (both hemispheres, y = 37). Red, green, and blue color scheme corresponds to lateral-medial (x), anterior-posterior (y), and superior-inferior (z) streamline trajectories, respectively. Primary olfactory cortical targets are labeled: AON, OT, FPC, TPC, AMY.

**Figure 4.**
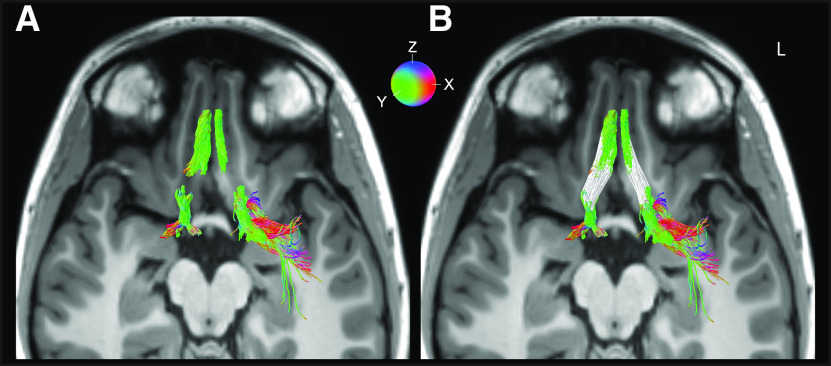
Example of natural cubic spline interpolation, overlaid on the subject's T1 image. ***A***, Streamlines generated using probabilistic tractography, seeding in the olfactory bulb, olfactory tract midpoint, and cortical olfactory regions. ***B***, The same streamlines as shown in ***A***, with the interpolated streamlines included (white). Red, green, and blue color scheme corresponds to lateral-medial (x), anterior-posterior (y), and superior-inferior (z) streamline trajectories, respectively.

Most importantly, bilateral continuous streamlines between the olfactory bulb and primary olfactory cortex were found in one subject (Fig. 3). In two other subjects, fiber group 1 overlapped with fiber groups 2 and 3 in the right hemisphere only. In many subjects, a small area of signal dropout near the sphenoid sinus prevented continuous tracking across the entire length of the olfactory tracts. In these subjects, fiber group 1 was separated from fiber groups 2 and 3 by a small gap. The length of fiber group 1, measured from the olfactory bulbs to the point of signal drop out, was: left hemisphere, mean 24.04 mm ± SD 4.81 mm; and right hemisphere, mean 27.12 mm ± SD 6.99 mm. The Euclidean distances between the posterior end of fiber group 1 and the anterior-most end of fiber groups 2 and 3 were: left hemisphere, mean 10.1 mm ± SD 4.1 mm; and right hemisphere, mean 8.5 mm ± SD 4.7 mm. In these subjects, we used a natural cubic spline interpolation method (Lee, 1989) to estimate the path of the olfactory tracts across the gap (Fig. 4).

Across subjects, streamlines in fiber group 1 projected posteriorly from the olfactory bulbs, following along the length of the olfactory sulci. Anterior projections of streamlines in fiber groups 2 and 3 followed along the olfactory sulcus and passed superiorly to the optic nerves before connecting to streamlines in fiber group 1, either directly or via interpolated segments. Posterior portions of streamlines in fiber groups 2 and 3 branched near the level of the optic chiasm to form the lateral, medial, and intermediate striae. Streamlines forming the intermediate striae curved sharply superiorly, entering AON gray matter, while those forming the lateral striae curved sharply laterally to meet FPC gray matter, and those forming the medial striae curved sharply medially to meet OT gray matter (Fig. 3*C*). Some streamlines of the lateral branch continued through the uncinate fasciculus to meet the TPC, and through temporal lobe white matter to meet AMY. Only one streamline identified in one subject reached the EC.

We quantified the connectivity of the olfactory tracts with each primary olfactory cortical region, defined as whether streamlines in fiber group 2 or 3 existed connecting the olfactory tract midpoint ROI with each cortical region. In all subjects, connectivity was present in at least one hemisphere between the olfactory tracts and the AON, the FPC, the TPC, and the OT. In 76% of subjects, connectivity with the AMY was also present in at least one hemisphere. Only one subject showed connectivity with the EC in the right hemisphere. Connection density, a measure of connection strength (Hagmann et al., 2008), was calculated for each connection by dividing the number of streamlines present by the volume (mm^3^) of the target cortical ROI. Group connectivity and connection density results are listed in Table 1. Individual subjects' connectivity and connection density results are listed in Table 2.

**Table 1.** Connectivity and connection densities of the olfactory tracts

	Hemisphere	Streamlines (median)	Streamlines IQR (Q3–Q1)	Connection density (mean ± SEM)	Number of subjects
AON	Left	271	301	0.77 ± 0.12	25
	Right	295	351	0.72 ± 0.11	24
FPC	Left	104	247	0.89 ± 0.16	22
	Right	15	54	0.22 ± 0.07	19
TPC	Left	173	289	1.01 ± 0.21	23
	Right	23	174	0.30 ± 0.08	18
OT	Left	451	359	1.70 ± 0.23	24
	Right	223	206	1.17 ± 0.19	24
AMY	Left	1	22	0.06 ± 0.03	14
	Right	2	36	0.03 ± 0.01	14
ENT	Left	0	0	0	0
	Right	0	0	0.4e–5	1

Columns depict the median and interquartile range [IQR (Q3 – Q1)] of the number of streamlines found between the olfactory tracts and the Regions of Interest (ROIs), the mean and Standard Error of the Mean (SEM) of connection density for each ROI, as well as the number of subjects in which the connectivity between the olfactory tracts and each ROI was identified.

**Table 2.** Individual streamlines and connection densities of the olfactory tracts

Subject	AON (#/density)		FPC (#/density)		TPC (#/density)		OT (#/density)		AMY (#/density)		ENT (#/density)	
	Left	Right	Left	Right	Left	Right	Left	Right	Left	Right	Left	Right
1	748/2.16	–	442/2.18	–	85/0.42	–	451/2.75	99/0.51	33/0.03	–	–	–
2	915/1.77	454/1.05	–	165/0.73	–	199/1.21	–	178/0.67	3/0.002	157/0.13	–	–
3	1/0.002	250/0.46	93/0.39	21/0.08	94/0.47	16/0.07	599/2.55	368/1.56	256/0.24	71/0.07	–	–
4	776/1.87	50/0.12	492/1.98	56/0.22	243/1.1	48/0.28	836/3.62	193/1.12	–	8/0.007	–	–
5	256/0.5	7/0.02	40/0.16	2/0.01	34/0.14	–	155/0.52	117/0.56	1/0.001	–	–	–
6	144/0.38	367/1.05	279/1.53	10/0.07	125/0.61	10/0.07	210/1.24	244/1.16	47/0.05	1/0.001	–	–
7	84/0.23	412/1.24	62/0.28	196/1.44	746/4.75	213/1.09	914/3.81	411/1.36	904/0.8	343/0.31	–	1/0.001
8	428/1.33	34/0.08	107/0.77	26/0.19	44/0.21	29/0.13	311/1.35	198/0.85	1/0.001	2/0.002	–	–
9	558/1.36	123/0.34	–	5/0.03	6/0.03	23/0.11	502/2.01	353/0.97	–	42/0.03	–	–
10	273/0.65	677/1.66	65/0.48	–	144/0.76	2/0.01	212/0.72	8/0.03	22/0.02	–	–	–
11	36/0.09	100/0.23	323/1.15	–	368/0.95	–	363/1.43	69/0.37	4/0.005	–	–	–
12	81/0.26	563/1.54	31/0.15	–	50/0.17	–	220/0.71	575/1.77	–	–	–	–
13	174/0.47	127/0.4	32/0.14	–	243/0.86	–	112/0.48	384/1.39	–	–	–	–
14	113/0.28	895/2.1	93/0.68	–	340/1.13	–	90/0.31	–	122/0.13	–	–	–
15	168/0.25	290/0.48	321/2.2	65/0.29	372/1.6	126/0.39	1269/3.59	536/2.59	–	4/0.004	–	–
16	373/0.8	315/0.71	293/1.98	89/0.7	499/2.01	241/0.92	531/2.03	309/0.96	146/0.13	2/0.002	–	–
17	570/2.11	215/0.79	186/1.19	102/0.36	454/2.24	188/0.65	54/0.2	865/2.59	–	–	–	–
18	36/0.13	64/0.22	462/2.73	42/0.36	572/2.59	224/1.04	569/2.4	206/1.14	21/0.02	78/0.1	–	–
19	386/0.92	518/0.94	–	2/0.01	–	12/0.02	309/0.98	23/0.08	–	–	–	–
20	147/0.22	78/0.13	240/1.59	16/0.1	173/0.76	59/0.23	781/2.54	346/1.38	–	19/0.02	–	–
21	346/0.94	307/1	116/0.63	3/0.02	229/1.01	–	499/1.88	1008/4.52	–	–	–	–
22	347/0.78	709/1.05	11/0.09	18/0.1	182/0.5	174/0.53	551/1.4	182/0.51	3/0.003	50/0.05	–	–
23	414/0.93	451/0.83	104/0.5	133/0.72	144/0.43	207/0.73	86/0.31	187/0.53	–	8/0.007	–	–
24	61/0.12	295/0.6	191/1.31	15/0.13	520/2.18	6/0.03	547/1.99	499/1.66	–	–	–	–
25	271/0.69	342/0.9	21/0.17	2/0.01	79/0.27	38/0.11	1068/3.6	223/0.9	3/0.003	36/0.04	–	–
Total *N*	25	24	22	19	23	18	24	24	14	14	0	1

Individual subjects' data. **#**: number of streamlines identified between the olfactory tracts and the Region of Interest (ROI); density: connection density for each ROI, defined as the number of streamlines divided by the ROI volume (mm^3^); total *N*: total number of subjects with connectivity identified between the olfactory tracts and the ROI. Each cell shows #/density; – denotes connections where no streamlines were found.

### The olfactory tract atlas

**Figure 5.**
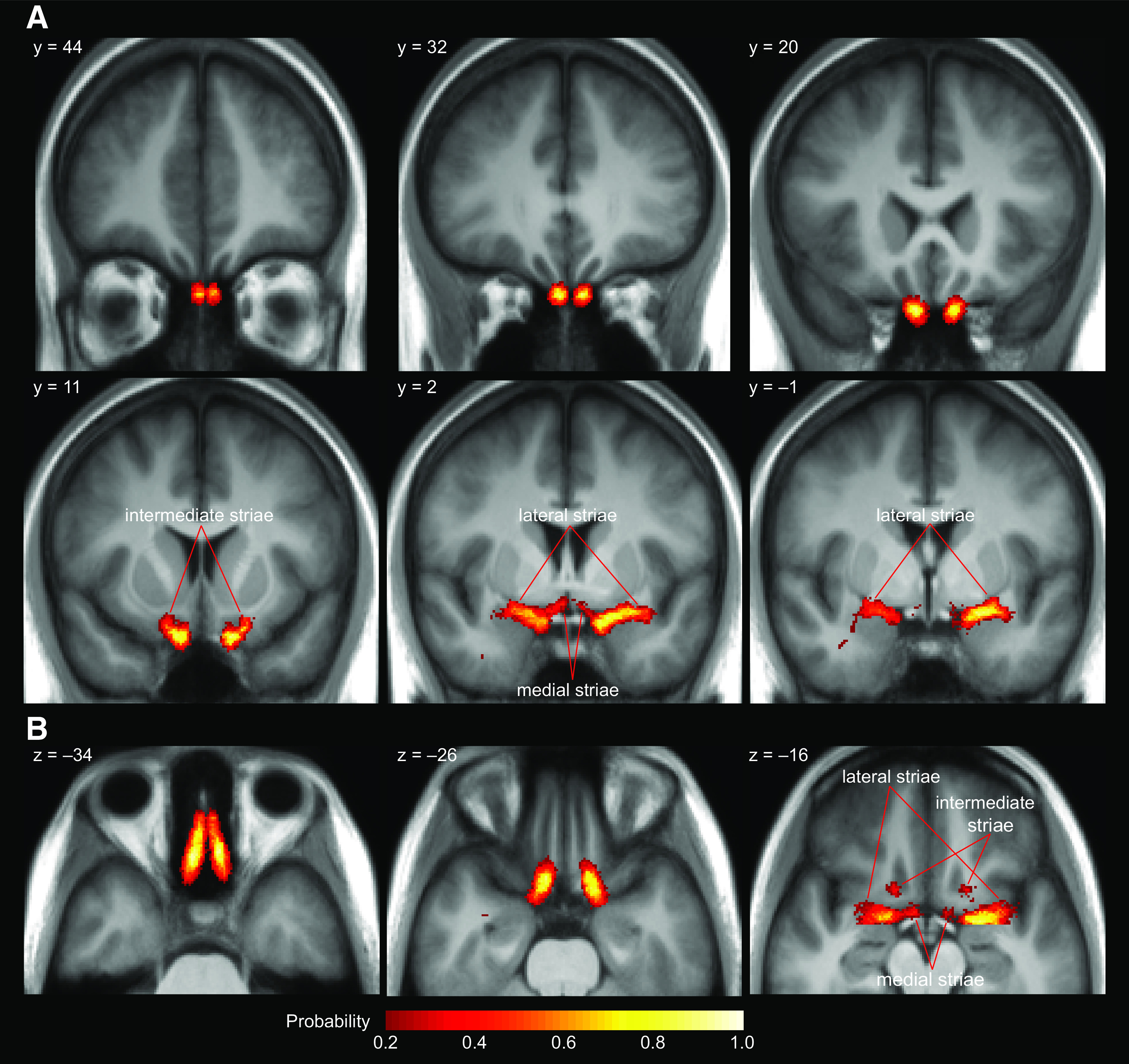
Probabilistic atlas of the olfactory tracts in MNI space. ***A***, Coronal slices showing the trajectory of the olfactory tracts from the bulbs (y = 44) to the superior projections of the intermediate striae (y = 11), and the projections of the medial and lateral striae (y = 2 and y = –1). ***B***, Axial slices showing the projections of the tracts from the bulbs (z = –34), the point where the tracts cross superiorly to the optic nerves (z = –26), and where all three striae are visible in each hemisphere (z = –16). Voxels overlapping in >20% of subjects are overlaid on a mean image of all subjects' MNI-normalized T1 images. This atlas is freely available on NeuroVault (https://neurovault.org/collections/ZTCWDMII/) and on BrainLife (https://brainlife.io/project/5ac2a489e182730027c55588).

Based on our tractography results, we created a normalized, probabilistic atlas to define the locations of the olfactory tracts in MNI space (Fig. 5). We created a binarized mask for each subject that consisted of voxels traversed by olfactory tract streamlines. We then transformed the masks into MNI space and averaged them across subjects to create a probability map of voxels traversed by the olfactory tracts. The atlas captures the trajectory of the olfactory tracts, as they project posteriorly, slightly superiorly and slightly laterally toward the primary olfactory cortex. The three branches of the olfactory tracts (i.e., lateral, medial, and intermediate striae) are clearly visible in the atlas in both hemispheres.

### Microstructure of the olfactory tracts

**Figure 6.**
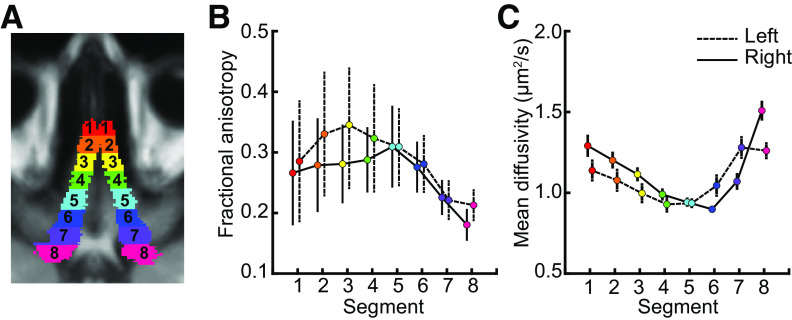
Diffusion microstructure profiles of the olfactory tracts. ***A***, Segments (1–8) of the olfactory tract atlases in each hemisphere in MNI space. ***B***, Fractional Anisotropy (FA) along the longitudinal axis of the olfactory tract. The FA values of each voxel were weighted by the probability of the olfactory tract atlas and averaged across all voxels for each segment. ***C***, Same as ***B*** but for Mean Diffusivity (MD).

Next, we used our probabilistic olfactory tract atlas to extract measures of microstructure integrity (i.e., FA and MD) from the olfactory tracts of individual subjects. We first divided the normalized atlas into eight anterior-posterior segments (6.25-mm width) in each hemisphere (Fig. 6*A*), and then transformed the segmented atlases into each subjects' native diffusion space and extracted the voxel-wise FA and MD values. Finally, we averaged the FA and MD values for each segment in each hemisphere across voxels, weighting FA and MD values by each voxel's probability value in the atlas, thus giving more weight to values closer to the core of the tract, and less weight to those near the edges of the tract that may have partial volume effects with surrounding cerebrospinal fluid. As expected, we found that FA and MD values varied by segment (Fig. 6*B*,*C*), presumably driven by local anatomic features.

### Tract microstructure integrity is related to olfactory function

**Figure 7.**
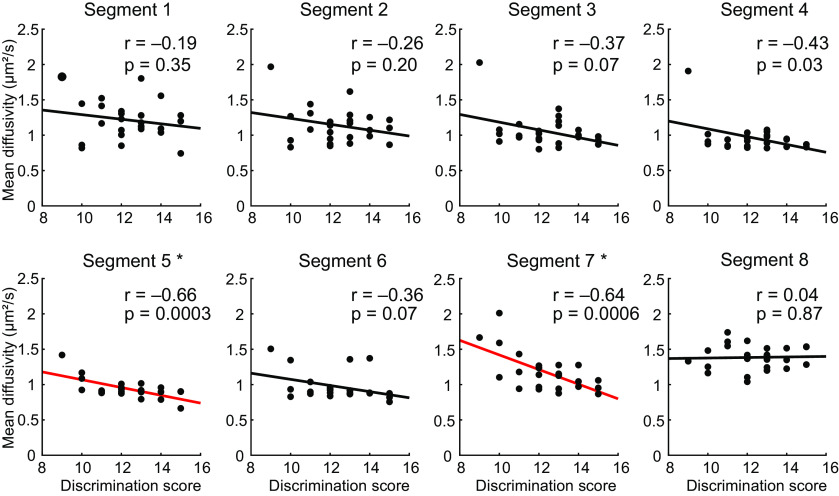
Pearson correlations between Mean Diffusivity in the olfactory tracts and olfactory discrimination scores. MD values were averaged across hemispheres for each segment. The straight line indicates least squares fit. Asterisks indicate statistically significant correlations (Bonferroni corrected for eight segments × three measures).

To test whether microstructure integrity in the olfactory tracts is relevant for olfactory perceptual function, we next tested correlations between the weighted mean FA and MD values for each segment (averaged across both hemispheres) and the three Sniffin' Sticks tests (threshold, discrimination, and identification). We found statistically significant correlations [Bonferroni corrected for multiple comparisons (eight segments × three measures)] between the MD values in segments 5 and 7 and the Sniffin' Sticks discrimination scores (Fig. 7). Both correlations were significant when controlling for sex (Bonferroni corrected; segment 5: b = –0.57, *p* = 0.0021; segment 7: b = –0.54, *p* = 0.0054) and age using multiple regression (Bonferroni corrected; segment 5: b = –0.65, *p* = 0.0004; segment 7: b = –0.62, *p* = 0.0012). We found no significant (Bonferroni corrected) correlations with FA, and no significant correlations between MD and the threshold or identification tests.

## Discussion

The likely cortical endpoints of the human olfactory tracts were first outlined nearly 70 years ago using silver myelin staining in *post mortem* brains (Allison, 1954). More recently, several groups have attempted to delineate these projections using modern dMRI methods *in vivo* (Skorpil et al., 2011; Fjaeldstad et al., 2017; Milardi et al., 2017). However, because of methodological limitations, these studies were unable to provide a comprehensive characterization of the striae and their cortical connectivity. In the present study, we implemented innovative imaging and tractography techniques to accomplish this goal. We identified the three striae of the olfactory tracts in 25 subjects, and discovered *in vivo* connectivity patterns matching those identified in *post mortem* data by Allison (1954). Based on these results, we have created the first publicly available probabilistic atlas of the olfactory tracts in MNI space. Additionally, we investigated microstructural properties of the tracts, and found that MD correlates with olfactory discrimination scores. In summary, our results provide the first comprehensive characterization of *in vivo* human olfactory tract connectivity, along with evidence for a relationship between olfactory tract microstructure and olfactory perceptual function.

In our data, the lateral, medial, and intermediate stria were identified in all subjects in at least one hemisphere. The lateral striae were the largest, and curved sharply laterally to meet FPC, TPC, and AMY. The medial striae curved medially to meet the OT, located at the base of the nucleus accumbens. The intermediate striae were the smallest, and projected superiorly to meet AON near the olfactory trigone. All three striae are clearly visible in both hemispheres within our probabilistic olfactory tract atlas.

We found reliable connectivity between the olfactory tracts and FPC, TPC, the AON, and the OT, with all subjects showing these connections in at least one hemisphere. In addition, 76% of subjects showed relatively sparse connectivity with the AMY in at least one hemisphere. This is consistent with Allison (1954)'s findings, wherein the majority of lateral striae fibers were found to reach FPC and TPC, with relatively few fibers continuing to meet AMY. Connectivity with EC, observed in both rodents and macaques (Haberly and Price, 1978a,b; Carmichael et al., 1994; Miyamichi et al., 2011), was nearly absent in our data. This could be because of one of two reasons. First, while Haberly and Price note connectivity with the entire extent of the lateral EC in the rodent, Carmichael and colleagues report that only Layer I of the rostral EC receives sparse olfactory tract inputs in the macaque, and Allison reports no olfactory tract connectivity with EC in the human. In both rats and macaques, association fibers between the EC and piriform cortex are much denser than fibers projecting directly between the EC and the olfactory bulb (White, 1965; Haberly and Price, 1978a,b; Carmichael et al., 1994). While the human EC is likely involved in olfactory processing (Poellinger et al., 2001; Bao et al., 2016, 2019), it may be two synapses away from the olfactory bulb rather than directly connected. Further investigation is warranted to determine the specific olfactory connectivity patterns of the human EC. Second, the lack of connectivity observed in our data may be because of known limitations with diffusion tractography methods. Tracking directly from the olfactory tracts to EC requires streamlines to cross piriform gray matter, where the diffusion signal tends to be more isotropic, and thus not conducive to tractography. Additionally, where direct streamlines are found, it is impossible to tell whether they represent direct synaptic connectivity with the olfactory bulb, or rather secondary synaptic connections with the piriform cortex. This may also explain the reduced number of subjects and the reduced density of streamlines found connecting the olfactory tracts with the AMY. Thus, diffusion tractography may not be an appropriate method for evaluating these particular connections. Further methodological innovation will be necessary to identify the presence or absence of these pathways in the human.

In addition to connectivity analyses, we characterized diffusion-based measures of tissue microstructure in the olfactory tracts. Fractional anisotropy (FA) and Mean Diffusivity (MD) are calculated from the diffusion signal and serve as noninvasive proxy measures of microstructural tissue properties, such as cell body or axon density, thickness of myelination, and the spatial organization of the underlying fiber architecture (Basser and Pierpaoli, 1996; Song et al., 2003, 2005). In segment 1, comprising the olfactory bulbs (gray matter), we found relatively low FA and relatively high MD values. In successive segments 2–5, comprised of the myelinated, single-trajectory core of the olfactory tracts, we see increasing FA and decreasing MD. Segments 6–8 comprise portions of the olfactory tracts that cross over the optic nerves and branch into several striae, including multiple fiber orientations and partial volume effects with neighboring gray matter. Accordingly, we see decreasing FA and increasing MD in these segments. FA and MD measures have been correlated with learning and skills training (Bengtsson et al., 2005; Scholz et al., 2009; Hofstetter et al., 2013), perceptual performance (Yeatman et al., 2011), and neurodegeneration-related loss of function (Song et al., 2003, 2005) in functionally-specific white matter pathways. An open question is whether olfactory tract microstructure is similarly related to olfactory perceptual performance. We observed significant correlations between odor discrimination scores and MD measures in segments 5 and 7 of the olfactory tracts, and most other olfactory tract segments showed similar nonsignificant trends. Differences between segments are likely because of varying noise levels along the lengths of the tracts, driven by magnetic field inhomogeneities, and partial volume effects with surrounding anatomic structures. However, the general direction of these effects suggests that tissue integrity in the human olfactory tracts supports olfactory perceptual function. We speculate that MD measures in the olfactory tracts may in part reflect individual variations in myelination or axon density, thus affecting the speed or bandwidth of olfactory information transfer. We note that our subject sample (25 healthy young adults who scored above anosmic thresholds) may be too limited to fully capture microstructure-function relationships. We suggest that future investigations include larger sample sizes, and consider wider age ranges, varied olfactory ability, and clinical populations with olfactory deficits.

In our study, we used modern technological innovations to provide a comprehensive characterization of human olfactory tract connectivity *in vivo*. Two previous dMRI studies (Skorpil et al., 2011; Fjaeldstad et al., 2017) attempted to reconstruct the olfactory tracts using the tensor model, and while they were able to reconstruct portions of the tracts, they were unable to characterize the branching and curving striae or the cortical connectivity of the tracts (Tournier et al., 2008). Our study and one previous study (Milardi et al., 2017) used a CSD model to address this issue. While Milardi and colleagues identified the larger lateral striae, they were unable to identify the intermediate and medial striae, likely because of a combination of susceptibility artifacts and low voxel resolution. In the present study, we applied an optimized RESOLVE sequence (Porter and Heidemann, 2009), designed specifically to reduce susceptibility artifacts and achieve a higher scanning resolution (1.5 mm) than has been used before to investigate the human olfactory system. Additionally, our subjects wore individualized head stabilizers during scanning to prevent motion. With these data, we were able to characterize all three striae of the olfactory tracts and identify their connectivity with primary olfactory cortex. We also provide the first in-depth description of the functionally-relevant microstructural properties of the tracts and their relationships with olfactory function.

While the RESOLVE sequence greatly improves image quality with relatively little susceptibility artifact, it requires a 7-fold increase in scan-time, making it less suitable for clinical settings. Additionally, we still observed a small region of signal drop-out near the sphenoid sinus in most subjects, preventing continuous tractography across the entire lengths of the olfactory tracts. However, based on postmortem observations, we are confident that interpolating between the two fiber groups accurately describes the trajectory of these white matter fibers. Additionally, when reconstructing the olfactory tracts, it is important to watch for streamline “jumping,” where streamlines may progress in anatomically impossible directions, especially in regions with low signal (Mori, 2007). When exclusionary ROIs were not placed to constrain tracking, we found that streamlines seeded in the olfactory bulb would jump into the parallel fibers of the gyrus rectus. To prevent such jumping resulting in erroneous streamlines, we placed extensive exclusionary ROIs in the gyrus rectus.

In summary, our results offer an in depth look at the *in vivo* anatomy of the human olfactory tracts. They provide the first step toward *in vivo* investigations of human olfactory tract structure-function relationships, which could be extended to address questions regarding microstructural changes following olfactory perceptual training (Haehner et al., 2013; Jiramongkolchai et al., 2021). In addition, our methods may be used in combination with our atlas to investigate olfactory tract integrity in clinical populations presenting with anosmia, such as those with Alzheimer's disease, Parkinson's disease, or multiple sclerosis.
